# Multiple endocrine neoplasia type 1 involving both the liver and lung: a case report

**DOI:** 10.1186/s12957-022-02622-1

**Published:** 2022-05-10

**Authors:** Jianlin Lai, Yangyang Huang, Junyi Wu, Hui Cheng, Funan Qiu

**Affiliations:** 1 Shengli Clinical Medical College of Fujian Medical University, Fuzhou, China; 2 Department of Hepatobiliary Surgery, Fujian Provincial Hospital, Fujian Medical University, Fuzhou, China; 3 Department of Pathology, Fujian Provincial Hospital, Fujian Medical University, Fuzhou, China

**Keywords:** Case report, Liver, Lung, Multiple endocrine neoplasia type 1, Genetic sequencing

## Abstract

**Background:**

Multiple endocrine neoplasia type 1 (MEN1) is a rare autosomal dominant tumor syndrome with a high degree of heterogeneity in clinical phenotypes, generally involving the parathyroid, anterior pituitary, and enteropancreas. In recent years, several new insights into the clinical features of MEN1 have been reported in the literature. However, it is not clear whether MEN1-associated primary tumors can occur in the liver.

**Case presentation:**

We report the case of a 52-year-old man with multiple endocrine neoplasia type 1 diagnosed by genetic sequencing. After uniportal thoracoscopic right middle lobectomy, laparoscopic radical resection of the liver tumors, and radiofrequency ablation of the parathyroid space, the parathyroid hormone level decreased from 177 pg/ml to a normal level (20 pg/ml). No local tumor recurrence was observed during a follow-up of 5 months.

**Conclusion:**

We report the first case of MEN1 with simultaneous liver and lung involvement in which the patient underwent radical resection of the tumors, and we propose the possibility that the liver and other nonendocrine organs may also develop diseases associated with MEN1; although, this view needs further verification. Gene detection has crucial clinical significance for guiding diagnosis and treatment.

## Background

MEN1 is a rare autosomal dominant tumor syndrome with a high degree of heterogeneity in clinical phenotypes. It has a substantial tendency towards neoplasm formation in two or more organs and often occurs in the parathyroid gland, enteropancreas and anterior pituitary [1, 2]. Since MEN1 syndrome was reported to be a genetic disease in 1997 [3], an increasing number of atypical MEN1 tumors, including but not limited to thymic and bronchial carcinoid tumors, breast tumors, and uterine tumors, have been found over the years [4–6]. However, a primary MEN1 tumor in the liver has not been reported. Moreover, in MEN1, a hereditary disease, there is no clear association between the type of gene mutation and clinical manifestations [7]. These unrepresentative clinical phenotypes make diagnosis difficult. Once misdiagnosed, the interests of the patient will be greatly affected. Hence, when the clinical manifestations are atypical, clinicians still need to be alert to the possibility of MEN1. We report the first case of MEN1 with simultaneous liver and lung involvement in which the patient underwent radical resection of the tumors, and we propose the possibility that primary tumors of the liver can also occur in MEN1; although, this view needs further verification.

## Case report

**Fig. 1:**
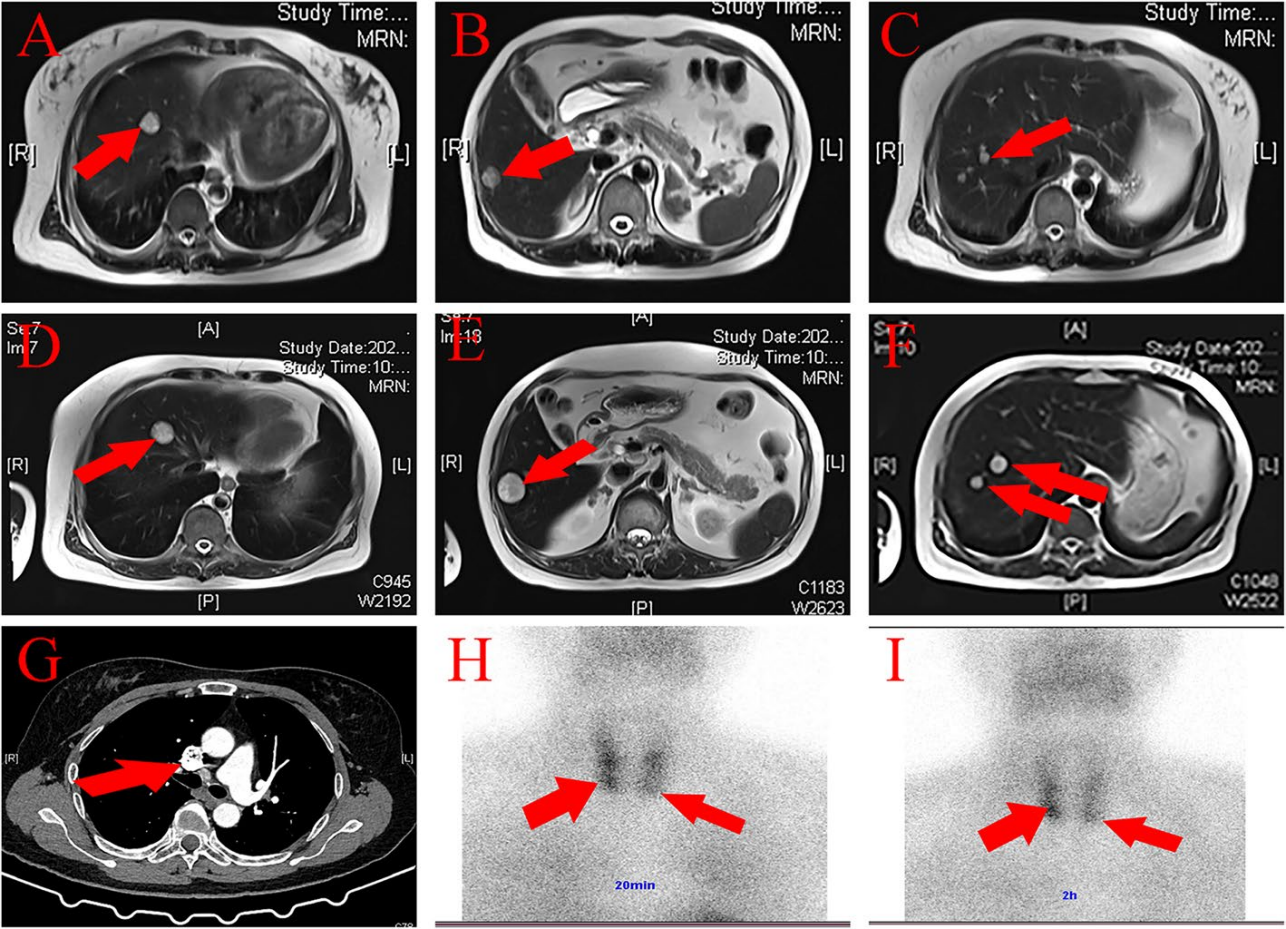
Clinical features before hepatectomy. The red arrows point to the location of lesions. **A**–**C** Multiple lesions were found in VI, VI, and VII segments with the largest diameter of about 1.8 cm by T2-weighted magnetic resonance image of liver in July 2020. **D**–**F** Multiple lesions in VI, VI, and VII segments were larger than before, with a largest diameter of about 2.9 cm in December, 2020. **G** In July 2020, computed tomography (CT) enhanced arterial phase showed the tumor, about 3.7 cm × 2.7 cm in size, located in the middle lobe of right lung and exhibited a inhomogeneous enhancement. **H**, **I** In December, 2020, Technetium-99m SESTAMIBI at 20 min and 2 h suggested parathyroid adenomas located in the lower poles of both sides

A 52-year-old male farmer was admitted to our hospital due to “liver space-occupying mass, cholecystolithiasis” by abdominal ultrasound on July 11, 2020. There were no clinical symptoms, such as abdominal pain, abdominal distension, acid regurgitation, jaundice, or rash. No enlarged thyroid gland or parathyroid gland were palpated during physical examination, and no obvious abnormality was found from more specific abdominal examinations. More than 20 years ago, “pituitary adenoma resection” was performed in a tertiary referral hospital in Fujian Province because of “pituitary adenoma.” The patient had a history of “kidney stone” for 5 years. He had neither a history of smoking nor drinking and had a good nutritional status according to nutritional risk screening tool 2002 (NRS-2002). In addition, 2 years ago, his mother underwent laparoscopic distal pancreatectomy for a “pancreatic tumor” in our hospital, and postoperative pathology revealed a pancreatic neuroendocrine tumor (G2). The imaging findings obtained in our hospital suggested metastatic tumors in segments IV and VI of the liver, multiple hemangiomas in segment VIII of the liver, central-type lung cancer of the middle lobe of the right lung, parathyroid adenoma, bilateral adrenal multiple adenomas, cholecystolithiasis with chronic cholecystitis, and right kidney stones (Fig. 1A–C, G). According to the above auxiliary examination, lung cancer with liver metastasis was considered as the initial diagnosis. Then, this patient was transferred to a tertiary referral hospital in Fujian Province, where ultrasound-guided fine-needle aspiration (US-FNA) cytology was performed on the parathyroid gland and lung and liver lesions; the results suggested lung adenocarcinoma with liver metastasis but interestingly, no tumor cells were found in the parathyroid mass. After 2 cycles of neoadjuvant chemotherapy (paclitaxel + lobaplatin), uniporal thoracoscopic right middle lobectomy plus mediastinal lymph node dissection was performed on October 14, 2020. The operation was smooth, and there were no obvious postoperative complications. Postoperative pathology showed atypical adenocarcinoma in the middle lobe of the right lung, a tumor thrombus in the vein, and no invasion into the pulmonary membrane or lymph nodes. The immunohistochemical results are as follows: CK(+); TTF-1(−); NapsinA(−); P63(−); P40(−); P53(−); CD56(+); CgA(+); Syn(+); Ki-67 5%(+); SSTR2(+); ATRX(+); and DAXX(+). Then, the patient was diagnosed with atypical adenocarcinoma with liver metastasis in the middle lobe of the right lung (pT2N0M1). Regrettably, MEN1 was not diagnosed when the patient was discharged from the hospital.

**Fig. 2:**
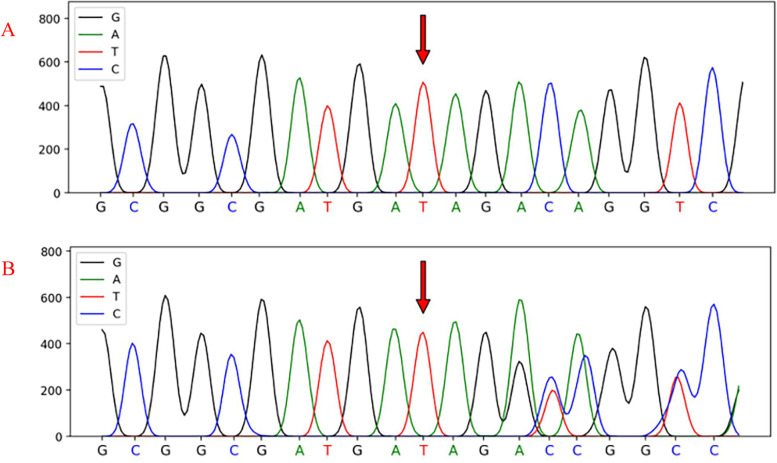
The result of genetic sequencing. **A** The reference genetic sequence of MEN1. **B** The genetic sequence of proposita showing mutations in chr11:64577329. Four base of mutation, GTCT respectively, were detected. **C** Pedigree chart of proposita and his first-degree relatives

**Fig. 3:**
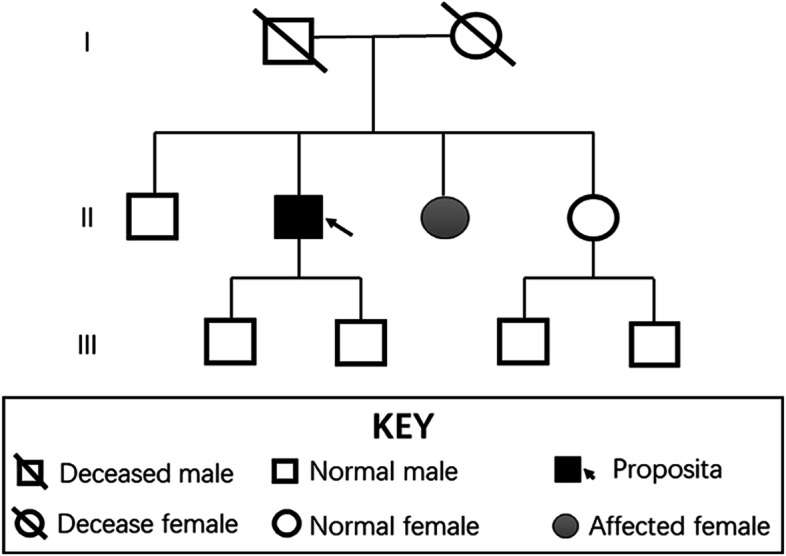
The family tree of the patient

The patient then revisited our centre on December 14, 2020. No abnormalities were found from the detailed physical examination, the blood routine test, serum biochemistry, and coagulation index assessment. However, liver MRI showed that the liver tumor was more advanced than before (Fig. 1D–F) and parathyroid adenoma was suggested by thyroid SPECT/CT (Fig. 1H, I). Various hormone indices were examined before the operation. The patient’s BMI was 25.39, CEA was 2.51 ng/ml (< 5), AFP was 2.61 ng/ml (< 7), PIVKA-II was 17.00 mAU/ml (< 40), and AFP-L3/AFP was less than 0.5. His serum parathormone was 177.00 pg/ml (reference interval (ref 15–88 pg/ml), serum calcium was 2.82 mmol/L (ref 2.11–2.52), serum phosphorus was 0.88 mmol/L (ref 0.85–1.51), adrenocorticotrophic hormone was 12.7 pg/ml (ref 7.2–63.6), renin (upright posture) was 8.90 ng/L (ref 4.00–24.00), angiotensin II (upright posture) was 185.99 ng/L (ref 49.00–252.00), angiotensin II (lying posture) was 160.43 ng/L (ref 25.00–129.00), aldosterone (upright posture) was 14.23 ng/dL (ref 4.00–31.00), aldosterone (lying posture) was 8.10 ng/L (ref 1.00–16.00), cortisol (8 am) was 262.32 ng/L (ref 240–680), cortisol (4 pm) was 170.97 nmol/L (ref < 276.00), sex hormone binding globulin was 55.91 nmol/L (ref 14.5–48.4), dehydroepiandrosterone was 58.50 μg/dL (ref 38–313), follicle-stimulating hormone was 3.51 IU/L (ref 1.27–19.26), luteinizing hormone was 1.46 IU/L (ref 1.25–8.63), prolactin was 6.54 ng/ml (ref 2.64–13.13), growth hormone was 0.02 ng/ml (ref 0.004–1.406), testosterone was 1.80 nmol/L (ref 6.07–27.24), progesterone was 0.86 ng/ml (ref 0.1–0.84), estradiol was 14.54 pg/ml (ref 15–38.95), plasma insulin was 9.93 mU/L (ref 2.6–24.9), c-peptide was 3.03 μg/L (ref 1.1–4.4), dopamine was 93.8 pmol/L (ref ≤ 195.7), adrenaline was 86.9 pmol/L (ref ≤ 605.4), norepinephrine was 958.6 pmol/L (ref 414–4435.5), and gastrin was 52.10 pg/ml (ref 13–115). Based on the obtained evidence, a clinical diagnosis of MEN1 was made. Blood samples were taken for genetic sequencing (Fig. 2), which indicated that the MEN1 gene had a deletion mutation of GTCT in chr11:64577329, resulting in the mutation of amino acid No. 85 from isoleucine to serine. Subsequently, an early termination signal was generated at the 33rd position of the new reading frame, causing normal protein function loss. Gene mutations of *TERT/ENG/BLM/POLE/MUTYH/NQO2/FANCC* were detected in the whole genome of the patient; however, these mutations have not been found to have clinical significance. At the same time, we conducted genetic tests on 7 first-degree relatives of the propositus and found that there was a germline mutation of *men1c.249-252del (p. ile855erfster33)*, which was the same as the mutation in the propositus (Fig. 3).

**Fig. 4:**
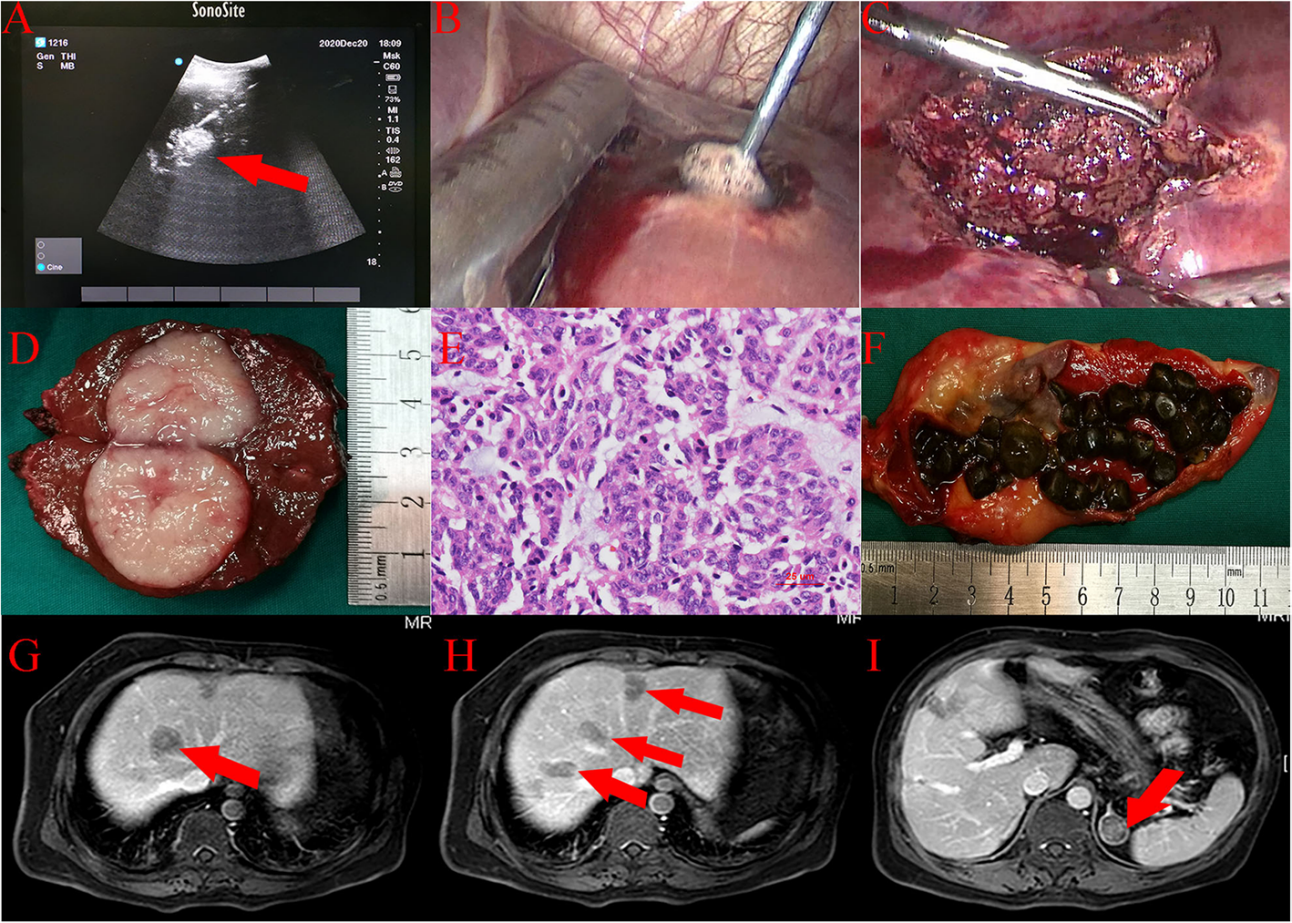
Clinical features of intraoperative and postoperative follow-up. The red arrows point to the location of lesions. **A** Laparoscopic ultrasound (LUS) showing high echo signal of the tumor in S4 segment of liver, which suggested that the tumor was degenerated under radiofrequency ablation (RF). **B** Endoscopic image of radiofrequency ablation in S4 segment of the liver. **C** Laparoscopic resection of liver mass in S4 segment. **D** Gross specimen of the tumor in S4 segment with a section size of about 3 × 3 cm, which had fish-flesh appearance. **E** Microscopic (histologic) image (HE × 40) suggested atypical carcinoid of S4 segment of the liver. **F** The gross specimen of cholecyst which was filled with gallstones. **G**, **H** Arterial phase of magnetic resonance imaging of the liver. No tumor recurrence was found in the operative area. The liver lesions in the area of radiofrequency ablation showed coagulative necrosis. **I** Arterial phase of magnetic resonance imaging. Adrenal space occupying lesion, 2.8 cm × 2.4 cm, with heterogeneous enhancement, was considered as adrenal adenoma. And there was no significant change in diameter of the tumor

**Fig. 5:**
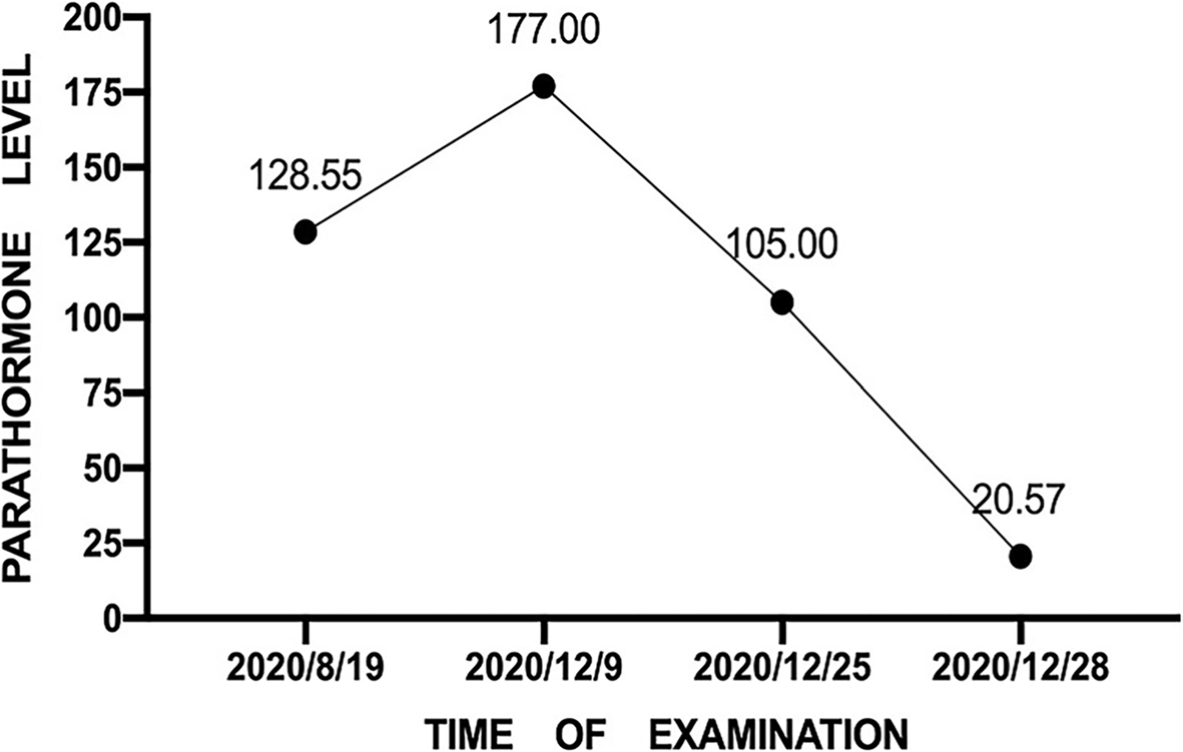
Change trend of parathyroid hormone in the course of disease

After multidisciplinary consultation (the Department of Endocrinology, Basic Surgery, Urology and Anasthesiology), radiofrequency ablation (RFA) of the right liver tumors (one in S6, two in S7 and one in S8), laparoscopic resection of the liver tumor in VI, endoscopic RFA of the liver tumors (one in S4, one in S5, and one in S6) and cholecystectomy were performed on December 24, 2020 (Fig. 4A–D, F). The operation was smooth, and the postoperative recovery was good. RFA of left and right lower pole parathyroid masses was performed on December 28, 2020 and December 29, 2020, respectively. Then, we performed a haematologic review of the patient. His parathyroid hormone was decreased to 20.57 pg/ml, serum calcium was 2.09 mmol/L, and serum phosphorus was 1.02 mmol/L, as expected (Fig. 5). Although the cell morphology was atypical under light microscopy, the combination of immunohistochemical results, CgA(+++); CD56(+++); Syn(+++); TTF(−); Ki67(15%+); NapsinA(−); CK(pan)(+++); CK18(+++); Heparl(−); CK19(+++); and Glypican3 (−), suggested atypical carcinoid of the liver (Fig. 4E). Then, the final diagnoses were as follows: multiple endocrine adenomatosis (MEN1); multiple atypical carcinoid in the liver; cholecystolithiasis with chronic cholecystitis; primary hyperparathyroidism; multiple parathyroid adenomas; right kidney calculi; and multiple adrenal adenomas. No local tumor recurrence was observed in the liver during a follow-up of 5 months, and there was no significant change in the size of the adrenal nodules (Fig. 4G–I).

## Discussion and conclusion

The MEN1 gene was first reported in 1977 [3], is located on chromosome 11q13 and is expressed in cells throughout the body, acting as an autosomal dominant tumor suppressor gene. At present, the theory of Knudson’s two-hit hypothesis is widely accepted as the hypothesized pathogenicity of this disease [8]. Homozygous mice with MEN1 gene deletion die in utero. In heterozygotes with a secondary somatic frameshift mutation, nonsense mutation, missense mutation, frame deletion or insertion, site splicing, or other mutation in the MEN1 allele that occurs after birth, the organs are likely to develop the disease. The diagnosis of MEN1 includes three criteria: (1) two or more MEN1-related primary endocrine tumors (pituitary, parathyroid or pancreas) in the same individual; (2) gene detection of MEN1 gene mutations; and (3) first-degree relatives of MEN1 patients with at least one MEN1-related tumor [2]. MEN1 can be diagnosed by satisfying any of the above criteria. In this case, this patient underwent transsphenoidal adenomectomy for a pituitary tumor 20 years ago and most recently, he was treated because of a “liver space-occupying mass.” Multiple space-occupying lesions of the whole body were found by imaging. Pathological biopsy showed that the liver and lung lesions were endocrine tumors. Genetic sequencing showed the *men1c.249-252del (p.ile855erfsTer33)* germline mutation, which could lead to a diagnosis of MEN1 both clinically and genetically. The incidence of this disease in nonendocrine organs is so low that clinicians do not pay enough attention to this clue. It was not until the patient returned to our hospital that MEN1 was considered after multidisciplinary consultation. Then, whole genome sequencing was performed for the patient, and 7 of his first-degree relatives were screened for the MEN1 gene.

As MEN1 is a hereditary disease, the importance of gene detection for diagnosis and treatment is self-evident. Genetic testing is recommended for the following situations [1, 2]: (1) two or more typical or atypical tumors related to MEN1; (2) parathyroid adenomas occurring before the age of 30 or multiple parathyroid diseases, multiple pancreatic neuroendocrine tumors or gastrinomas at any age; and (3) first-degree relatives of the patient as well as those in the pedigree diagnosed clinically or genetically. It has been confirmed that the survival duration of patients with MEN1 gene mutations is shorter than that of patients without gene mutations. Once a gene mutation is found, close clinical follow-up observation should be carried out according to established guidelines to achieve timely detection, timely diagnosis, timely treatment, and improve survival time. First-degree relatives of such patients are also recommended for MEN1 gene screening to identify which family members need to enter the clinical observation process. More importantly, early detection of highly malignant lesions will increase the R0 resection rate of the tumors, helping to achieve a longer survival duration [7, 9, 10]. One patient with the same MEN1 gene mutation as the proband was found in this pedigree, and there was no clinical phenotype at present. Regular imaging and haematological follow-up of the proband and his MEN1 gene mutation-positive relatives will help improve the quality of life and survival time of the patients.

At present, MEN1-related endocrine adenomas are treated based on the strategies for sporadic endocrine adenomas, and radical resection is the first choice for treatment. However, the guidelines do not clearly point out differentiation between and treatment strategies of primary and metastatic lesions found in multiple tumors at the same time. Even for highly malignant intestinal tumors, the 5-year survival rate can reach 80% with radical resection of the intestinal primary and metastatic lesions. The perioperative mortality was less than 5%. Furthermore, to improve endocrine syndrome-related symptoms and improve patient quality of life, it is feasible to perform cytoreductive surgeries to improve the overall survival and reduce the endocrine level of 95% of patients [11–13].

In our case, we carried out genetic testing on the patient in time (though not at the first visit) and confirmed the existence of a MEN1 gene mutation. Minimally invasive liver tumor resection combined with intraoperative liver radiofrequency ablation (RFA) was used to preserve the healthy liver tissue as much as possible. Percutaneous radiofrequency ablation of parathyroid tumors not only ensured a radical effect but also prevented the patient from undergoing too many major spinal surgeries in a short time, thus avoiding negative psychological consequences.

No scholars have suggested that MEN1-related primary tumors might occur in the liver. Although liver metastasis may occur in MEN1-related duodenopancreatic tumors [14] and thymic tumors [15], metachronous liver metastasis has been reported in only MEN1-related lung tumors [16]. No liver metastasis has been reported in the pituitary tumor or parathyroid tumor. In our case, the discovery of liver and pulmonary tumors was synchronous, and the lesions were radically resected. As mentioned above, the level of parathyroid hormone returned to normal after uniportal thoracoscopic right middle lobectomy, liver lesion resection and RFA of the parathyroid glands. Moreover, no local tumor recurrence was observed during a follow-up of 5 months. To the best of our knowledge, there have been no similar reports.

The biggest limitation is that it is not completely clear whether the nature of the lung and liver tumors were multifocal primary tumors or metastases. However, most MEN1-related tumors are benign, and a characteristic of MEN1 is that tumors can in multiple organs and appear as multiple lesions all over the body. Moreover, liver and lung biopsies of the patient showed that the tumors belonged to a highly differentiated type, meaning that the probability of distant metastasis was relatively low [17], and no new lesions were found after 5 months of follow-up. We put forward the hypothesis that the liver and lung may have multifocal primary tumors at the same time. Furthermore, we propose the possibility that primary tumors of the liver can also occur in MEN1; although, this view needs further verification.

In this patient, SPECT/CT found that there was a hypermetabolic mass in each of the bilateral inferior thyroid poles, and the possibility of bilateral inferior parathyroid adenoma was considered because of the increases in parathyroid hormone level to 177 pg/ml and serum calcium. Therefore, primary hyperparathyroidism (PHPT) could be diagnosed. At present, the optimal timing and scope of parathyroid surgery are still controversial. According to the guidelines, more than 3.5 thyroid glands should be removed in such patients [2], but the possibility of permanent parathyroid dysfunction after extensive parathyroid surgery is as high as 40%. Severe hypercalcaemia and parathyroid carcinoma are rare in patients with MEN1-related PHPT, and the main goal of treatment is control of parathyroid hormone levels. When the number of enlarged parathyroid glands is less than 2, the scope of parathyroidectomy can be minimized as much as possible, and individualized treatment can be realized [18, 19]. It has been confirmed that ultrasound-guided radiofrequency ablation of parathyroid adenoma is effective and safe [18, 20]. The return of parathyroid hormone (20 pg/ml) to a normal range due to a decrease in parathyroid hormone (177 pg/ml) after the operation suggested that the treatment was effective and that there were no obvious complications after the operation. A limitation is that the parathyroid hormone level was not re-examined during follow-up.

Abdominal MRI also found multiple adrenal adenomas. However, haematological analysis results did not suggest aldosteronism or hypercortisolemia. However, no increased adrenal metabolism was found on PET-CT, so continued follow-up was needed. Surgical resection can be considered when symptoms occur, when the tumors obviously grow or are larger than 4 cm [2].

The patient recovered well after the surgical treatments. He said that he would strictly follow the doctor's advice, adding, “I will also urge relatives who carry pathogenic genes to undergo clinical follow-up”. Both doctors and the patient are confident in achieving high-quality and long-term survival in the future.

In summary, the clinical phenotypes of MEN1 come in a variety of forms, and tumors in atypical-related organs such as the liver and lung endocrine are easy to miss diagnosis of this disease. When this disease is suspected, we should carry out comprehensive laboratory tests, imaging examinations, gene detection and other screening approaches. Furthermore, multidisciplinary consultation is recommended when MEN1 is suspected to reduce the rate of missed diagnosis. The final goal is to increase survival time and improve quality of life through timely discovery, timely diagnosis, and timely treatment.

## Data Availability

All data generated or analyzed during this study are included in this published article.

## Abbreviations

MEN1: Multiple endocrine neoplasia type 1
NRS-2002: Nutritional risk screening tool 2002
US-FNA: Ultrasound-guided fine-needle aspiration
MRI: Magnetic resonance imaging
SPECT/CT: Single-photon emission computed tomography-computed tomography
CEA: Carcinoembryonic antigen
AFP: α-fetoprotein
PIVKA-II: Protein induced by vitamin K absence or antagonist-II
RFA: Radio-frequency ablation
PHPT: Primary hyperparathyroidism
PET-CT: Positron emission tomography-computed tomography
